# Covariates and Spatial Interpolation of HIV Screening in Mozambique: Insight from the 2015 AIDS Indicator Survey

**DOI:** 10.3390/ijerph17165630

**Published:** 2020-08-05

**Authors:** Pascal Agbadi, Jerry John Nutor, Ernest Darkwah, Henry Ofori Duah, Precious Adade Duodu, Robert Kaba Alhassan, Kimberly Baltzell

**Affiliations:** 1 Department of Nursing, College of Health Sciences, Kwame Nkrumah University of Science and Technology, Kumasi, Ghana; pascalagbadi@gmail.com; 2 Department of Family Health Care Nursing, School of Nursing, University of California, 2 Koret Way, Suite N431G, San Francisco, CA 94143, USA; Kimberly.Baltzell@ucsf.edu; 3 Department of Psychology, University of Ghana, P.O. Box LG 84, Legon, Ghana; edarkwah@ug.edu.gh; 4 Research Department, FOCOS Orthopaedic Hospital, Accra, Ghana; duahh@focosgh.com; 5 East Surrey Hospital, Canada Avenue, Redhill, Surrey RH1 5RH, England, UK; precious.duodu@nhs.net; 6 School of Nursing and Midwifery, University of Health and Allied Sciences, Ho, Ghana; ralhassan@uhas.edu.gh

**Keywords:** HIV screening, HIV/AIDS, spatial interpolation, Mozambique, sub-Saharan Africa

## Abstract

We examined the factors associated with human immunodeficiency virus (HIV) screening and developed a HIV screening prevalence surface map using spatial interpolation techniques to identify the geographical areas with the highest and lowest rates of HIV screening in Mozambique. We analyzed the cross-sectional 2015 Mozambique AIDS Indicator Surveys with an analytic sample of 12,995 participants. Analyses were conducted on SPSS-21, STATA-14, and R freeware 3.5.3. We adjusted for the sample design and population weights. Results indicated that 52.5% of Mozambicans had undergone HIV screening. Mozambicans with these characteristics have a higher probability of undergoing HIV screening: females, those with a primary education or higher, urban dwellers, residents of wealthy households, having at least one lifetime sexual partner, and dwelling in these provinces—Niassa, Tete, Manica, Sofala, Inhambane, Gaza, Maputo Provincia, and Maputo Cidade. The spatial map revealed that the national and regional estimates mask sub-regional level estimates. Generally, zones with the highest HIV screening prevalence are found in southern provinces while the lowest prevalence was found in the northern provinces. The map further revealed intraregional differences in HIV screening estimates. We recommend that HIV screening be expanded, with equitable screening resource allocations that target more nuanced areas within provinces which have a low HIV screening prevalence.

## 1. Introduction

Despite decades of global concerted efforts to combat the spread of human immunodeficiency virus (HIV) and mitigate its effects on the global population, HIV infections still rank among some of the most important public health concerns across the world. According to the Joint United Nations Programme on HIV/AIDS (UNAIDS), an estimated 1.7 million new HIV infections were recorded worldwide in 2018 alone [1]. Epidemiological research shows the geographical and contextual differences in infection rates across different regions of the world, with countries in the global south being the most affected [2,3]. For example, sub-Saharan Africa has consistently topped the list of regions with the highest infection rate [2], with nations in the southern part of Africa bearing the brunt of the HIV epidemic [4]. Coupled with the myriad of other problems including poverty, tropical diseases such as malaria, rampant corruption, and armed conflicts that have bedeviled the continent for decades, the HIV epidemic continues to wreak havoc on the life expectancy, development, and quality of life of the African population.

To combat the HIV burden, research scientists and epidemiologists often recommend a ramping up of screening or testing as a crucial first step. Screening is done in pre-symptomatic and/or asymptomatic populations to identify and treat those who are yet to show signs [5] and is crucial for gaining an important early situational awareness about the disease’s severity, tendencies and transferability. These, in turn, are important in predicting the likely effectiveness of interventions [5,6,7]. Rapid screening has also been shown to be the main means for individuals to be informed about their HIV status. Therefore, screening is a critical first step in the global fight against the HIV epidemic.

In 2014, UNAIDS and partners launched the 90–90–90 targets. The aim was to have 90% of all HIV-positive persons diagnosed, provide antiretroviral therapy (ART) for 90% of those diagnosed, and, for 90% of ART recipients, achieve viral suppression by 2020 [8]. The most important aim is step one, to identify 90% of all HIV positive people. However, in a recent systematic analysis of national HIV treatment cascades from 69 countries by Levi and colleagues [9], none of the countries had achieved the 90–90–90 targets. They reported that diagnosis (target one—90% of all HIV-positive people diagnosed) ranged from 87% (the Netherlands) to 11% (Yemen) [9].

Mozambique ranks among countries with the highest burden of HIV, with a prevalence of 12.6% in adults between the ages of 15 and 49 years. In 2018 alone, there were 150,000 new infections and 54,000 HIV deaths in the country [1]. Globally, this small south-eastern African country ranks as the fifth highest HIV infected country in the world according to 2015 estimates [10]. Over the years, researchers have listed relatively little knowledge about HIV, poor health systems, and inadequate access to HIV preventive and therapeutic services among a host of other factors as key contributors to the country’s high HIV prevalence [11,12,13,14]. However, factors that influence HIV screening in Mozambique and other sub-Saharan African countries have not been well studied.

In the face of the evidence and in response to recommendations, the government of Mozambique, with support from its aid partners, have invested heavily in the procurement of HIV screening and diagnostic testing equipment [7]. To support these positive steps and their consequent gains, insights into the specific trends and discrepancies in screening prevalence and associated predictors are necessary for providing empirical guidance for the continuous evaluation, prioritization and direction of ongoing HIV response activities. However, such insights are largely missing in the body of literature on HIV diagnosis and prevalence in Mozambique. Therefore, this study aimed to elucidate differences in the screening prevalence within provinces in Mozambique using the most current nationally representative AIDS Indicator Survey data.

## 2. Materials and Methods

### 2.1. Design and Study Sample

The study used publicly available data from the cross-sectional Mozambique AIDS Indicator Survey (M-AIS) conducted in 2015. The study had two main objectives, to identify the characteristics of those who are more likely to be tested for HIV, and then to identify the geographical areas where testing rates varied. The M-AIS data is collected based on a two-stage sampling design. A master sample plan consisting of 307 primary sampling units (PSUs) was randomly selected from the 2007 Mozambique General Population and Housing Census sample frame. The 307 PSUs were stratified by the locality of residence, with 134 urban PSUs and 173 rural PSUs. From each PSU, twenty-four (24) households were selected, resulting in a total household sample size of 7368 households. The estimated number of individuals to be enumerated were 14,343, but a total of 13,032 individuals was reached, a response rate of 90.86%. Thirty-seven (37) cases did not have complete information on the outcome and the explanatory variables, therefore, they were removed from the dataset. The dataset used for this study contained 12,995 cases.

### 2.2. Measures

#### 2.2.1. Outcome

The outcome variable for the study is HIV screening. In the dataset, the variable with information on HIV screening is labelled as “Ever been tested for HIV” with a “Yes” or “No” response. The response “Yes” was coded as “1” and “No” as “0”.

#### 2.2.2. Explanatory Variables

The following ten sociodemographic variables were selected from the dataset as potential predictors: gender, age, education, marital status, religion, total lifetime sexual partners, history of sexually transmitted infections (STIs) 12 months before the survey, household wealth index, rural–urban place of residence, and region of residence. The household wealth index was already computed by the DHS program and available in the dataset. DHS used a principal component analysis (PCA) to assign weights to each asset in each household and cumulative scores were calculated from the assigned weights. From the PCA results, households were categorized into five quantiles: poorest, poorer, middle, rich, and richest. The computation used the following information: household characteristics (source of drinking water, type of toilet, sharing of toilet facilities, the main material for the roof, walls and floors, and type of cooking fuel amongst others household characteristics) and household possessions and assets (ownership of television, radio, vehicle, bicycles, motorcycles, watches, agricultural land, farm animals/livestock, and bank account, amongst others).

### 2.3. Data Analysis

Descriptive statistics and a test of association were performed in SPSS, and the multivariable analysis was performed in STATA. The analyses adjusted for the sample design (PSU and sample strata) and weights. The statistical significance threshold was pegged at the 5% level of significance (*p* ≤ 0.05). SPSS-21, STATA-14, and the R freeware 3.5.3 were used for the analyses.

The first objective of this study was to identify the characteristics of those who are more likely to be tested for HIV. In SPSS, the steps for adjusting for the complex sample procedure is reported elsewhere [15]. Sample characteristics were described using frequencies and percentages. A contingency table with chi-square test of independence was used to measure the association between the outcome variable and each explanatory variable. Explanatory variables that were significantly associated with the outcome were included and assessed in a multivariable model. We assessed the interaction effect of gender on the relationship between each study covariate and HIV screening among Mozambicans by computing, for each covariate, a complex samples Poisson model. A Wald chi-square test was performed to assess the significance of the interaction term.

In STATA, the “svy” command in a default mode was used to set up the analytic environment before analysis. The default “svy” computes the standard errors by using the linearized variance estimator called the first-order Taylor linearization. This procedure eliminated the incorrect estimation of the standard errors (SEs) associated with the confidence intervals of the regression coefficients. A Poisson regression was used to estimate the prevalence ratios. We checked the assumption of the overdispersion of the data using the nbreg command, and found that the likelihood-ratio (LR) test of alpha = 0 and the *p*-value of the LR test was greater than 0.05, implying that the conditional variance is equal to the conditional mean, making Poisson an appropriate model to fit for the outcome.

#### 2.3.1. Spatial Interpolation of HIV Prevalence

The second objective of this paper was to understand the HIV epidemic and inform programs and interventions at a lower geographical-level. The 2015 M-AIS has information on the HIV screening prevalence for 306 clusters. HIV screening prevalence at a cluster level through a spatial interpolation technique was performed using cluster-level geolocation data (longitude and latitude). The prevR package in the R freeware was used for the spatial interpolation [16]. The package is designed to determine the prevalence of outcomes from surveys with a stratified two-stage sample design [16]. Using functions available in the prevR package came with preinstalled functions which we used to generate the surface map for the HIV screening prevalence for Mozambique by adopting the Gaussian kernel estimator approach, with adaptive bandwidths for an equal number of persons surveyed [16]. A comprehensive methodology on how the prevR package can be used to obtain the surface map is reported elsewhere [16]. We also use the following packages in R for the analysis: foreign, maptools and ggplot2. We also generated a provincial HIV screening prevalence stratified by gender using the Quantum Geographic Information System (QGIS).

#### 2.3.2. Ethical Consideration

The Ethical Review Committee of the Mozambique Ministry of Health’s National Institute of Health and the Institutional Review Board of ICF International reviewed and approved the 2015 Mozambique AIS protocol [17]. The 2015 Mozambique AIS data is publicly available upon a simple registration-access request, so we did not seek for further ethical clearance. The data can be obtained from the DHS website at https://dhsprogram.com/data/dataset_admin/index.cfm.

## 3. Results

### 3.1. Sample Characteristics

The proportion of the respondents who had ever tested for HIV was 52.5% (Table 1). There were more women (59.5%) than men (40.5%) in the study population (Table 1). The majority of the respondents had attained a primary level of education (52.8% (Table 1)). The majority of them were currently married (64.9%), Muslims (39.3%), and residing in rural areas (63.3% (Table 1)).

### 3.2. Chi-Square Test of Independence between the Covariates and the Outcome

A chi-square test of independence and bivariate logistic regression analyses were performed to ascertain the relationship between the variables and ever screened for HIV. The results revealed that all the variables considered in the study were significantly associated with being tested for HIV. The proportion of females (61.3%) who had ever undergone a HIV screening was more than males (39.6%), and the proportion of urban dwellers (63.6%) screened was more than rural dwellers (46.1% (Table 1)). The proportion of post-secondary education holders (89.1%) who had undergone HIV screening was higher than those with a secondary (64.7%), primary (49.1%), and no education (43.3%) (Table 1). More people had undergone HIV testing in the richest households (69.6%) compared to the richer (61.5%), middle (48.5%), poorer (40.7%), and the poorest households (36.8% (Table 1)). A detailed description of the chi-square test of association results is presented in Table 1.

### 3.3. Sociodemographic and Sexual Behavioural Factors Regressed on HIV Screening Status

The adjusted complex samples Poisson regression revealed that the following sociodemographic factors are statistically significant predictors of HIV infection in Mozambique: sex, age, education level, marital status, total lifetime sexual partners, household wealth index, urban/rural residence, and region of residence (Table 2).

Mozambicans with these characteristics have a higher probability of undergoing a HIV screening: females, those with primary education or higher, urban dwellers, residents of wealthy households, having at least one lifetime sexual partner, and dwelling in these provinces—Niassa, Tete, Manica, Sofala, Inhambane, Gaza, Maputo Provincia, and Maputo Cidade.

### 3.4. Gender Modifies the Association between Each Covariate and HIV Screening

Gender modified the association between HIV screening and the following study covariates: respondent’s age (*p* < 0.001), education (*p* < 0.001), marital status (*p* < 0.001), religious affiliation (*p* < 0.001), total lifetime sexual partners (*p* < 0.001), STI status in the past 12 months (*p* < 0.001), household wealth (*p* < 0.001), rural/urban residence (*p* < 0.001), and province of residence (*p* < 0.001) (Table 3).

### 3.5. Drivers of HIV Testing in a Gender-Stratified Multivariate Model

The drivers of HIV testing are largely the same for both males and females in Mozambique. However, there are key differences in the way certain factors relate to HIV testing between men and women (Table 4). For instance, the effect of the total number of lifetime sexual partners on HIV testing, though the relationship goes in the same direction, was much bigger for women than for men (Table 4). Another important factor is education. The results indicate that the effect of education on HIV testing is slightly bigger for men than for women, although the relationship goes in the same direction (Table 4).

### 3.6. Spatial Map of Mozambique Showing Provincial HIV Screening Prevalence by Gender

**Figure 1:**
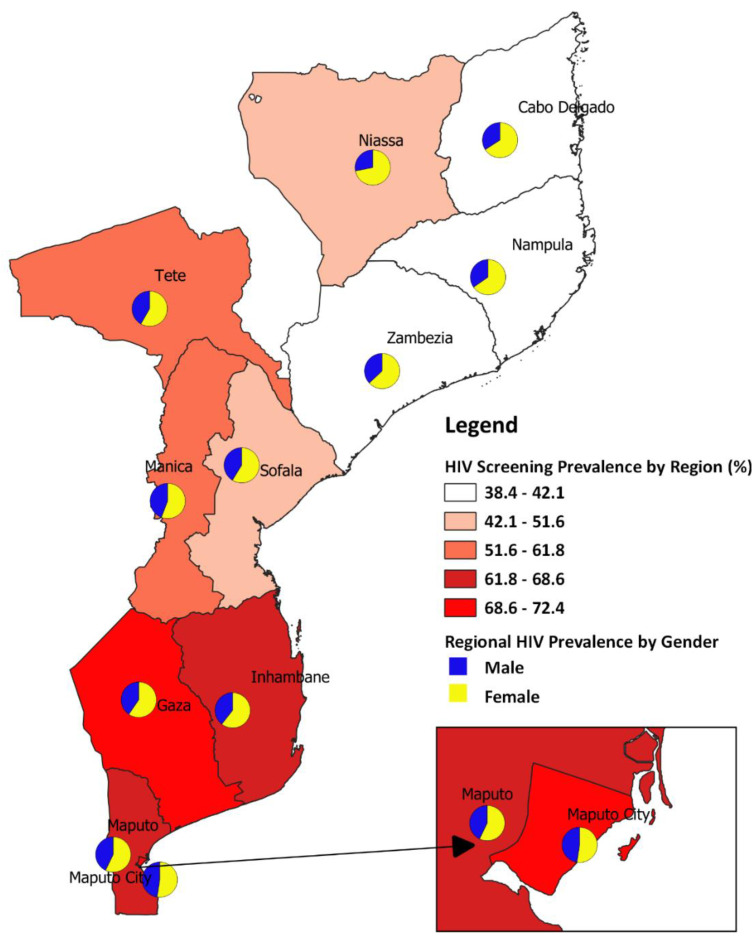
Provincial HIV screening prevalence by gender.

The national HIV screening prevalence for Mozambique was 52.5%. We stratified the HIV screening prevalence by province and discovered that the national estimate masked the prevalence at the provincial level (Figure 1; Table 1). For instance, Gaza’s estimate of 72.4% is higher than the national estimate and Nampula’s estimate of 38.4% is lower than the national estimate (Figure 1; Table 1). We further stratified the provincial HIV screening prevalence by gender represented with a pie graph on the map (Figure 1). The results indicate that the proportion of women who had undergone HIV screening was more than the proportion of men in all the eleven provinces (Figure 1 and Table S1).

### 3.7. HIV Screening Prevalence in Mozambique Estimated by the Kernel Estimator Approach

**Figure 2:**
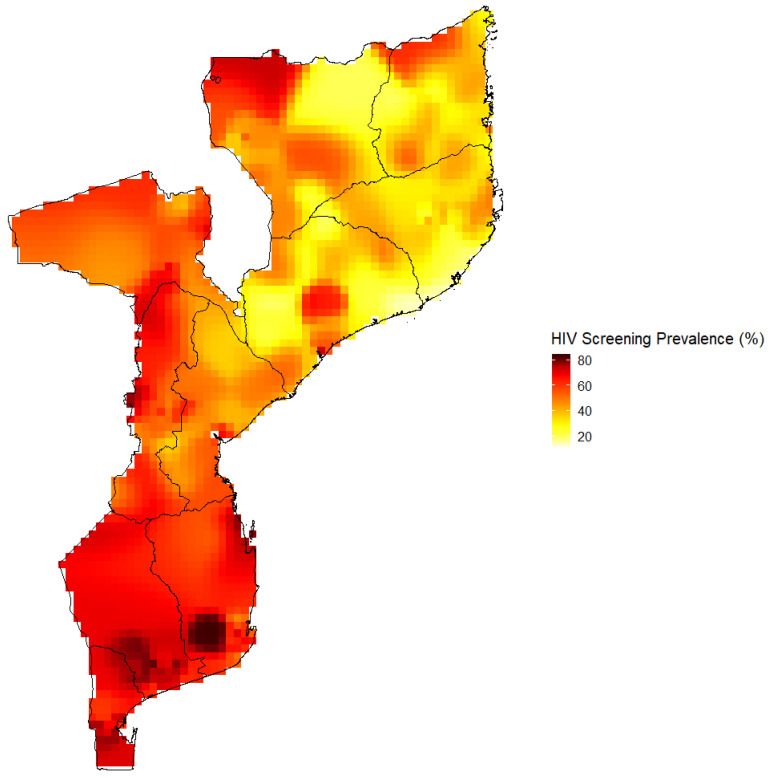
HIV screening prevalence in Mozambique estimated by the kernel estimator approach.

Overall, the HIV screening surface map revealed that national and regional level estimates mask sub-regional level estimates (Figure 2). The general observation is that the zones with the highest HIV screening prevalence are found in southern provinces and zones with the lowest prevalence are found in the northern provinces (Figure 2). Furthermore, the surface map revealed intraregional level differences in the HIV screening estimate. For instance, there are areas within Gaza with higher screening prevalence than others.

## 4. Discussion

HIV screening is critical to treatment, care and prevention. Adequate HIV screening is integral to meeting the 90–90–90 targets made by the UNAIDS [8]. Our study sought to estimate the prevalence of HIV screening and its associated factors among Mozambicans in their reproductive age using the 2015 M-AIS dataset. To eliminate the masking of sub-regional estimates that characterized national and regional level analyses, we developed an HIV/AIDS screening prevalence surface map using spatial interpolation techniques to identify geographical areas with the highest and lowest HIV screening rates in Mozambique. Our results emphasize the importance of geographical-level variations and the impact of factors including gender, marital status, age, education, wealth index and place of residence on HIV screening. We found that being female, being presently or previously married, having at least a primary education, living in an urban area, and coming from a wealthy home were linked to a higher likelihood of being screened for HIV. Although some of these factors have previously been linked to HIV screening [18,19], their geographical-level distributions and contributions have not been studied in Mozambique.

We found that a little over half (52.5%) of Mozambicans at reproductive age had undergone HIV screenings. The screening rate found in our study was higher when compared to the same population in Chad (42%), Burkina Faso (41%), and Sierra Leone (34%) but were lower than those found in Malawi (89%), Rwanda (95%) Zambia (82%) and Zimbabwe (89%) [20]. The high rates of HIV screenings in these countries were attributed to the mandatory HIV testing at prenatal clinics and mobile clinics for HIV screening [20]. However, our spatial interpolation revealed that there are areas in Mozambique with extremes of HIV screening prevalence compared to the national and regional estimates. For example, in Niassa province, the regional HIV screening prevalence ranged between 42.1% and 51.6%, however, the spatial analysis identified some clusters to be as low as 20% and some as high as 70%. Our findings also confirm the importance of applying spatial interpolation in population-based studies to unmask hidden details which can help design targeted interventions.

The spatial heterogeneity in HIV testing can be attributed to both the offer of HIV testing, i.e., the accessibility, and the demand for HIV testing (i.e., knowledge, attitudes, and beliefs regarding HIV and testing). HIV testing happen in healthcare facilities in Mozambique, however a greater disparity exists in terms of the access to healthcare facilities across the eleven provinces in a walking-to-healthcare facility scenario [21]. About 90% of Mozambicans were not within a walking distance of 60 min. For Maputo city, about 70% of the area are within an hour’s walking distance to a healthcare facility. However, the situation is worse for Tete, Cabo Delgado, and Gaza, with over 90% of the area outside of an hour’s walking distance to a healthcare facility [21]. Besides, a comprehensive knowledge of HIV varied across the provinces in Mozambique [22], and lower HIV knowledge and a fear of HIV related stigma prevented Mozambicans from accessing HIV testing services [23].

We also found that, compared to all age groups, adolescents between ages 15 and 19 years tend to have the lowest likelihood of getting screened for HIV. This result is particularly important because of the high rate of new infections among this age group [24] and the broad opportunity for implementing HIV prevention strategies during this critical period in life. Addressing adolescents’ lack of HIV testing to improve treatment and prevention requires an increase in community-based approaches to testing, youth friendly services, integrating HIV testing with other health services, such as reproductive health services, and building differentiated service models amongst others [25,26]. Additionally, a plethora of evidence suggests that school-based sex education interventions is linked with a sound HIV knowledge which is put into practice among adolescents and youths [14,27,28]. Therefore, school-based HIV knowledge, attitudes, and practice interventions can be pursued in Mozambique to promote HIV testing among adolescents.

The higher rates of HIV screening among women in the sample are possibly related to the mandatory screening during a prenatal clinic visit to prevent mother-to-child transmission [29]. Similarly, the higher rates of HIV screening among currently and previously married people could be explained by mandatory screening being required by some religious groups, especially Christians, in some parts of sub-Saharan Africa before performing marriage ceremonies [30,31,32]. These mandatory HIV screenings have been previously reported to help the uptake of HIV testing [29,32]. This suggests that a collaboration between public health officials and religious organizations may improve the uptake of HIV screening which could help prevent the spread of the virus. Besides, the higher rates of female screening compared to males also raises questions as to the relative impact/influence that HIV screening education campaigns could be having on the sexes. By stratifying our multivariate model by gender, our results highlight that risk perception regarding having multiple lifetime sexual partners is likely lower in men than women. Our findings basically confirmed a well-documented fact that men and boys in general have a lower likelihood than women to screen for HIV, know their status, and access and adhere to HIV treatment when they test positive [33]. This challenge has been attributed to certain gender norms that make the adoption of safer sex practices and seeking and accessing health services seem unmanly. These results suggest that the government and nongovernmental organizations in Mozambique must adopt policies and programs that encourage men to utilize health services frequently. Regarding HIV testing, community-based testing and counselling, a focus should be made on door-to-door service provisions, mobile outreaches, couples or male partner testing, and integrating HIV testing into existing sexual, reproductive and other health services [33].

Additionally, we found that people who are more educated, live in urban areas and who are from wealthy homes are more likely to get screened. The role of socioeconomic factors as a driver of HIV testing has been quite well-documented in sub-Saharan African countries [34,35]. These findings underscore the importance of attracting the most vulnerable and at-risk populations to HIV screenings. This could be done through targeted outreach programs, such as mobile clinics, and integrating HIV screening into routine healthcare services, improving home-based and self-screening and subsidizing the cost of the screening. Community leaders, such as chiefs, should also be involved in promoting HIV testing.

## 5. Strengths and Limitations

The study made use of a large, nationally representative survey dataset that is grounded in standardized methodology for analyses. Secondly, the study employed spatial interpolation techniques that have advantages over standard statistical techniques to identify the geographical variations of HIV screening prevalence in Mozambique. Additionally, our study uncovered the at-risk population and location of low HIV screening areas in Mozambique. These findings could serve as a framework for public health officials to design targeted interventions to increase HIV screening.

Our findings, however, are subject to limitations that must be taken into consideration. It is important to note that all the variables in this study, including HIV screening history, were self-reported. Like all self-reported data, some of the responses might be subjected to a social desirability bias. Additionally, we were not able to ascertain the actual reasons for participant’s prior HIV testing and therefore this was not accounted for in our analysis. Despite these limitations, this study has provided profound insights from a population-level survey analysis, as well as a spatial analysis of HIV screening prevalence in Mozambique for informed public health action.

## 6. Conclusions

In conclusion, this study could help public health and health policy officials to develop an effective intervention in Mozambique by showing where and towards which populations HIV screening resources should be allocated. We found that 52.5% of the population in Mozambique have been screened for HIV—with females, those with a primary education or higher, urban dwellers and those from wealthy homes more likely to be screened for HIV. An expansion of HIV screening, outreach through mobile clinics, home-based approaches and self-testing, wide-ranging coverage through outreach programs, community-based approaches, and integrating opportunities to be screened during regular medical care are critical for reaching all HIV-positive persons in sub-Saharan Africa with lifesaving treatments.

## Figures and Tables

**Table 1:** Weighted summary statistics and chi-square test of the independence between covariates and the outcome (*n* = 12,995).

Characteristics		Ever Screened for HIV		χ^2^, *p*-Value
		No	Yes	
	*n* (% ^a^)	*n* (% ^b^)	*n* (% ^b^)	
		6172 (47.5)	6823 (52.5)	
Gender				χ^2^ = 592.90, *p* ≤ 0.001
Male	5263 (40.5)	3180 (60.4)	2083 (39.6)	
Female	7732 (59.5)	2992 (38.7)	4740 (61.3)	
Age				χ^2^ = 811.95, *p* ≤ 0.001
15–19	2587 (19.9)	1733 (67.0)	854 (33.0)	
20–24	2271 (17.5)	865 (38.1)	1407 (61.9)	
25–29	1776 (13.7)	595 (33.5)	1181 (66.5)	
30–34	1532 (11.8)	583 (38.1)	949 (61.9)	
35–39	1391 (10.7)	534 (38.4)	857 (61.6)	
40–44	1118 (8.6)	536 (47.9)	582 (52.1)	
45–49	933 (7.2)	499 (53.4)	435 (46.6)	
50+	1386 (10.7)	828 (59.7)	558 (40.3)	
Education				χ^2^ = 461.91, *p* ≤ 0.001
No formal education	2744 (21.1)	1555 (56.7)	1189 (43.3)	
Primary	6857 (52.8)	3488 (50.9)	3369 (49.1)	
Secondary	3110 (23.9)	1098 (35.3)	2012 (64.7)	
Post-Secondary	284 (2.2)	31 (10.9)	253 (89.1)	
Marital status				χ^2^ = 314.23, *p* ≤ 0.001
Never married	2744 (21.1)	1713 (62.4)	1031 (37.6)	
Currently married	8435 (64.9)	3702 (43.9)	4733 (56.1)	
Previously married	1816 (14.0)	757 (41.7)	1058 (58.3)	
Religion				χ^2^ = 335.55, *p* ≤ 0.001
Catholic	3949 (30.4)	2069 (52.4)	1880 (47.6)	
Protestant Christians	2545 (19.6)	1459 (57.3)	1086 (42.7)	
Islam	5113 (39.3)	1934 (37.8)	3178 (62.2)	
No religion/others	1389 (10.7)	710 (51.2)	678 (48.8)	
Total lifetime sexual partners				χ^2^ = 650.17, *p* ≤ 0.001
0	851 (6.6)	737 (86.5)	115 (13.5)	
1	3770 (29.0)	1527 (40.5)	2244 (59.5)	
2	2770 (21.3)	1217 (43.9)	1553 (56.1)	
3–4	2653 (20.4)	1163 (43.8)	1490 (56.2)	
5–9	1450 (11.2)	776 (53.5)	674 (46.5)	
10+	786 (6.0)	382 (48.6)	404 (51.4)	
Undisclosed	713 (5.5)	371 (52.0)	343 (48.0)	
Had any STI last 12 months				χ^2^ = 18.83, *p* ≤ 0.001
No	12,506 (96.2)	5987 (47.9)	6519 (52.1)	
Yes	489 (3.8)	185 (37.8)	304 (62.2)	
Household wealth index				χ^2^ = 842.83, *p* ≤ 0.001
Poorest	2431 (18.7)	1536 (63.2)	895 (36.8)	
Poorer	2460 (18.9)	1459 (59.3)	1001 (40.7)	
Middle	2388 (18.4)	1230 (51.5)	1158 (48.5)	
Richer	2576 (19.8)	992 (38.5)	1584 (61.5)	
Richest	3140 (24.2)	956 (30.4)	2184 (69.6)	
Rural/Urban Residence				χ^2^ = 372.84, *p* ≤ 0.001
Urban	4769 (36.7)	1736 (36.4)	3034 (63.6)	
Rural	8226 (63.3)	4437 (53.9)	3789 (46.1)	
Province				χ^2^ = 864.34, *p* ≤ 0.001
Niassa	705 (5.4)	374 (53.1)	330 (46.9)	
Cabo Delgado	1304 (10.0)	780 (59.8)	525 (40.2)	
Nampula	2787 (21.4)	1718 (61.6)	1069 (38.4)	
Zambézia	1473 (11.3)	854 (57.9)	620 (42.1)	
Tete	906 (7.0)	389 (42.9)	517 (57.1)	
Manica	929 (7.2)	355 (38.2)	574 (61.8)	
Sofala	1222 (9.4)	591 (48.4)	631 (51.6)	
Inhambane	838 (6.4)	294 (35.1)	544 (64.9)	
Gaza	1059 (8.1)	292 (27.6)	767 (72.4)	
Maputo Provincia	838 (6.5)	263 (31.4)	575 (68.6)	
Maputo Cidade	933 (7.2)	262 (28.1)	671 (71.9)	

^a^ column percentage reported; ^b^ row percentage reported.

**Table 2:** Complex samples Poisson regression results of predictors of ever testing for HIV (*n* = 12,995).

Characteristics	PR [95% CI of PR]	APR [95% CI of APR]
Gender		
Male	1	1
Female	1.55 [1.47, 1.64]	1.56 [1.49, 1.65]
Age		
15–19	1	1
20–24	1.88 [1.73, 2.04]	1.37 [1.27, 1.48]
25–29	2.01 [1.86, 2.19]	1.46 [1.34, 1.58]
30–34	1.88 [1.71, 2.06]	1.40 [1.27, 1.53]
35–39	1.87 [1.70, 2.05]	1.43 [1.31, 1.58]
40–44	1.58 [1.42, 1.75]	1.25 [1.13, 1.38]
45–49	1.41 [1.07, 1.38]	1.10 [1.00, 1.20]
50+	1.22 [1.07, 1.38]	0.96 [0.85, 1.08]
Education		
No formal education	1	1
Primary	1.13 [1.05, 1.22]	1.19 [1.11, 1.28]
Secondary	1.49 [1.38, 1.62]	1.50 [1.38, 1.63]
Post-Secondary	2.06 [1.88, 2.25]	1.86 [1.69, 2.06]
Marital status		
Never married	1	1
Currently married	1.49 [1.39, 1.61]	1.35 [1.27, 1.42]
Previously married	1.55 [1.42, 1.70]	1.30 [1.21, 1.40]
Religion		
Islam	1	1
Catholic	1.12 [0.99, 1.26]	1.04 [0.95, 1.14]
Protestant Christians	1.46 [1.32, 1.61]	1.06 [0.97, 1.15]
No religion/others	1.14 [1.00, 1.30]	0.98 [0.88, 1.09]
Total lifetime sexual partners		
0	1	1
1	4.42 [3.66, 5.34]	3.04 [2.51, 3.67]
2	4.17 [3.44, 5.04]	2.88 [2.39, 3.47]
3–4	4.17 [3.46, 5.03]	2.94 [2.44, 3.54]
5–9	3.46 [2.83, 4.22]	2.90 [2.39, 3.51]
10+	3.82 [3.10, 4.71]	3.35 [2.73, 4.11]
Undisclosed	3.57 [2.82, 4.52]	2.89 [2.34, 3.56]
Had any STI last 12 months		
No	1	1
Yes	1.19 [1.10, 1.29]	1.04 [0.97, 1.12]
Household wealth index		
Poorest	1	1
Poorer	1.11 [1.00, 1.22]	1.07 [0.98, 1.17]
Middle	1.32 [1.18, 1.47]	1.19 [1.08, 1.32]
Richer	1.67 [1.52, 1.84]	1.31 [1.19, 1.45]
Richest	1.89 [1.72, 2.07]	1.37 [1.22, 1.54]
Rural/Urban Residence		
Rural	1	1
Urban	1.38 [1.30, 1.47]	1.10 [1.01, 1.19]
Province		
Cabo Delgado	1	1
Niassa	1.17 [0.97, 1.40]	1.20 [1.01, 1.41]
Nampula	0.95 [0.79, 1.15]	0.95 [0.82, 1.11]
Zambézia	1.05 [0.83, 1.32]	1.05 [0.88, 1.27]
Tete	1.42 [1.19, 1.69]	1.43 [1.23, 1.67]
Manica	1.53 [1.29, 1.83]	1.42 [1.21, 1.65]
Sofala	1.28 [1.08, 1.53]	1.18 [1.03, 1.36]
Inhambane	1.61 [1.35, 1.92]	1.46 [1.24, 1.78]
Gaza	1.80 [1.54, 2.10]	1.56 [1.36, 1.78]
Maputo Provincia	1.70 [1.46, 1.99]	1.31 [1.15, 1.49]
Maputo Cidade	1.79 [1.53, 2.09]	1.31 [1.15, 1.49]

PR: prevalence ratio; APR: adjusted prevalence ratio; CI: confidence intervals.

**Table 3:** Covariates of HIV screening among Mozambicans by gender (male/female).

	Females (*n* = 7732)	Males (*n* = 5263)	
	PR [95% CI of PR]	PR [95% CI of PR]	Wald Chi-Square Test
Age			F(15, 271) = 49.75, *p* < 0.001
15–19	1	1	
20–24	1.75 [1.60, 1.90]	2.14 [1.79, 2.56]	
25–29	1.75 [1.60, 1.91]	2.73 [2.29, 3.27]	
30–34	1.74 [1.58, 1.91]	2.35 [1.95, 2.59]	
35–39	1.58 [1.42, 1.75]	2.61 [2.15, 3.17]	
40–44	1.37 [1.23, 1.52]	2.22 [1.77, 2.77]	
45–49	1.16 [1.02, 1.32]	2.16 [1.76, 2.66]	
50+	0.92 [0.80, 1.05]	2.15 [1.72, 2.68]	
Education			F( 7, 279) = 120.00, *p* < 0.001
No formal education	1	1	
Primary	1.28 [1.19, 1.37]	1.35 [1.06, 1.71]	
Secondary	1.56 [1.44, 1.69]	2.31 [1.80, 2.97]	
Post-Secondary	1.91 [1.75, 2.08]	3.68 [2.85, 4.75]	
Marital status			F( 5, 281) = 72.35, *p* < 0.001
Never married	1	1	
Currently married	1.41 [1.30, 1.53]	1.38 [1.23, 1.54]	
Previously married	1.32 [1.19, 1.46]	1.48 [1.26, 1.72]	
Religion			F(7, 279) = 50.23, *p* < 0.001
Islam	1	1	
Catholic	1.11 [0.99, 1.24]	1.12 [0.91, 1.38]	
Protestant Christians	1.33 [1.21, 1.46]	1.66 [1.37, 2.01]	
No religion/others	1.14 [0.99, 1.31]	1.31 [1.05, 1.62]	
Total lifetime sexual partners			F(13, 273) = 37.91, *p* < 0.001
0	1	1	
1	5.87 [4.42, 7.80]	2.06 [1.55, 2.73]	
2	4.92 [4.46, 7.87]	2.23 [1.71, 2.92]	
3–4	6.23 [4.70, 8.26]	2.61 [2.03, 2.04]	
5–9	5.81 [4.24, 7.95]	2.58 [1.98, 3.35]	
10+	5.49 [3.58, 8.43]	3.15 [2.41, 4.12]	
Undisclosed	6.71 [4.91, 9.16]	2.59 [1.89, 3.55]	
Had any STI last 12 months			F(3, 283) = 102.39, *p* < 0.001
No	1	1	
Yes	1.30 [1.20, 1.41]	1.15 [0.97, 1.35]	
Household wealth index			F(9, 277) = 68.30, *p* < 0.001
Poorest	1	1	
Poorer	1.12 [1.01, 1.24]	1.16 [0.97, 1.40]	
Middle	1.29 [1.15, 1.45]	1.52 [1.26, 1.82]	
Richer	1.58 [1.43, 1.75]	2.06 [1.75, 2.42]	
Richest	1.68 [1.52, 1.86]	2.74 [2.32, 3.24]	
Rural/Urban Residence			F(3, 283) = 153.70, *p* < 0.001
Rural	1	1	
Urban	1.30 [1.22, 1.38]	1.68 [1.51, 1.88]	
Province			F(21, 265) = 29.22, *p* < 0.001
Cabo Delgado	1	1	
Niassa	1.24 [1.06, 1.45]	0.94 [0.62, 1.43]	
Nampula	0.93 [0.80, 1.09]	0.96 [0.63, 1.46]	
Zambézia	0.98 [0.77, 1.25]	1.11 [0.73, 1.70]	
Tete	1.25 [1.07, 1.47]	1.74 [1.17, 2.58]	
Manica	1.32 [1.13, 1.53]	2.01 [1.37, 2.94]	
Sofala	1.14 [0.99, 1.32]	1.55 [1.05, 2.28]	
Inhambane	1.44 [1.26, 1.65]	1.82 [1.22, 2.71]	
Gaza	1.59 [1.41, 1.78]	2.08 [1.41, 3.01]	
Maputo Provincia	1.52 [1.34, 1.71]	2.20 [1.09, 3.17]	
Maputo Cidade	1.46 [1.30, 1.65]	2.57 [1.78, 3.72]	

**Table 4:** Complex sample Poisson regression results of the predictors of having ever tested for HIV stratified by gender.

	Females	Males
	APR [95% CI of APR]	APR [95% CI of APR]
Age		
15–19	1	1
20–24	1.31 [1.21, 1.42]	1.75 [1.44, 2.12]
25–29	1.33 [1.23, 1.45]	2.19 [1.74, 2.74]
30–34	1.32 [1.20, 1.45]	2.05 [1.63, 2.59]
35–39	1.25 [1.14, 1.38]	2.49 [1.96, 3.16]
40–44	1.07 [0.96, 1.20]	2.23 [1.73, 2.87]
45–49	0.91 [0.81, 1.02]	2.11 [1.66, 2.69]
50+	0.73 [0.64, 0.84]	2.02 [1.56, 2.61]
Education		
No formal education	1	1
Primary	1.20 [1.12, 1.28]	1.27 [1.04, 1.56]
Secondary	1.33 [1.23, 1.44]	1.93 [1.54, 2.41]
Post-Secondary	1.49 [1.35, 1.65]	2.29 [1.83, 2.87]
Marital status		
Never married	1	1
Currently married	1.24 [1.17, 1.31]	1.21 [1.08, 1.36]
Previously married	1.24 [1.15, 1.34]	1.09 [0.92, 1.28]
Religion		
Islam	1	1
Catholic	1.09 [0.99, 1.19]	0.96 [0.82, 1.13]
Protestant Christians	1.08 [0.98, 1.18]	1.05 [0.90, 1.21]
No religion/others	1.04 [0.92, 1.18]	0.88 [0.74, 1.05]
Total lifetime sexual partners		
0	1	1
1	5.16 [3.90, 6.84]	1.45 [1.10, 1.91]
2	4.88 [3.69, 6.46]	1.53 [1.18, 1.99]
3–4	4.94 [3.74, 6.52]	1.55 [1.18, 2.04]
5–9	4.74 [3.49, 6.44]	1.52 [1.17, 1.96]
10+	4.21 [2.87, 6.16]	1.75 [1.32, 2.30]
Undisclosed	5.16 [3.81, 6.99]	1.39 [1.05, 1.84]
Had any STI last 12 months		
No	1	1
Yes	1.07 [1.00, 1.14]	1.06 [0.91, 1.25]
Household wealth index		
Poorest	1	1
Poorer	1.08 [0.98, 1.19]	1.06 [0.89, 1.26]
Middle	1.18 [1.06, 1.31]	1.26 [1.06, 1.50]
Richer	1.25 [1.13, 1.38]	1.48 [1.25, 1.75]
Richest	1.28 [1.14, 1.43]	1.70 [1.38, 2.09]
Rural/Urban Residence		
Rural	1	1
Urban	1.14 [1.06, 1.22]	1.02 [0.89, 1.18]
Region		
Cabo Delgado	1	1
Niassa	1.23 [1.07, 1.42]	1.02 [0.70, 1.48]
Nampula	0.92 [0.81, 1.03]	0.97 [0.67, 1.40]
Zambézia	0.97 [0.79, 1.19]	1.14 [0.79, 1.64]
Tete	1.23 [1.06, 1.43]	1.85 [1.29, 2.66]
Manica	1.20 [1.05, 1.39]	1.80 [1.28, 2.54]
Sofala	1.06 [0.93, 1.20]	1.37 [1.00, 1.89]
Inhambane	1.32 [1.16, 1.50]	1.70 [1.20, 2.40]
Gaza	1.40 [1.25, 1.57]	1.93 [1.41, 2.66]
Maputo Provincia	1.18 [1.06, 1.32]	1.47 [1.09, 1.98]
Maputo Cidade	1.11 [0.99, 1.24]	1.67 [1.23, 2.27]

## Supplementary Materials

The following are available online at https://www.mdpi.com/1660-4601/17/16/5630/s1, Table S1: Regional HIV Screening Prevalence (%) by Gender (*n* = 12,995).
