# Supplementary material for: Covariates and Spatial Interpolation of HIV Screening in Mozambique: Insight from the 2015 AIDS Indicator Survey
Source: Int J Environ Res Public Health. 2020 Aug 5;17(16):5630. doi: 10.3390/ijerph17165630 (PMC7460053; doi:10.3390/ijerph17165630)
Supplement: Supplementary file 1 [file ijerph-17-05630-s001.pdf]

Table S1. Regional HIV Screening Prevalence (%) by Gender (n = 12,995).

		Gender		
**Region (n)**		**Male**	**Female**	**p-value**
	**HIV Screening**	**n (%)**	**n (%)**	
Cabo Delgado (1304)	Yes	153 (26.5)	372 (51.2)	p ≤ 0.001
	No	424 (73.5)	355 (48.8)	
Niassa (705)	Yes	76 (24.9)	254 (63.5)	p ≤ 0.001
	No	229 (75.1)	146 (36.5)	
Nampula (2787)	Yes	298 (25.4)	771 (47.8)	p ≤ 0.001
	No	877 (74.6)	841 (52.2)	
Zambézia (1473)	Yes	168 (29.4)	451 (50.1)	p ≤ 0.001
	No	404 (70.6)	449 (49.9)	
Tete (905)	Yes	160 (45.8)	356 (64.0)	p ≤ 0.001
	No	189 (54.2)	200 (36.0)	
Manica (929)	Yes	196 (53.3)	378 (67.4)	p ≤ 0.001
	No	172 (46.7)	183 (32.6)	
Sofala (1222)	Yes	198 (41.1)	434 (58.6)	p ≤ 0.001
	No	284 (58.9)	307 (41.4)	
Inhambane (838)	Yes	140 (48.1)	404 (73.9)	p ≤ 0.001
	No	151 (51.9)	143 (26.1)	
Gaza (1059)	Yes	195 (55.1)	572 (81.1)	p ≤ 0.001
	No	159 (44.9)	133 (18.9)	
Maputo Provincia (838)	Yes	225 (58.1)	350 (77.6)	p ≤ 0.001
	No	162 (41.9)	101 (22.4)	
Maputo Cidade (933)	Yes	274 (68.0)	397 (74.9)	p ≤ 0.001
	No	129 (32.0)	133 (25.1)	
